# The prognostic value of copeptin for acute intracerebral hemorrhage patients

**DOI:** 10.3892/etm.2012.804

**Published:** 2012-11-07

**Authors:** AIMEI ZHANG, JUN LI, XIAOYUN LI, LI SONG, HONGFANG LI

**Affiliations:** 1 Department of Neurology, Affiliated Hospital of Ji Ning Medical University, Jining 272029;; 2 Graduate School of Tianjin Medical University, Tianjin 300070, P.R. China

**Keywords:** copeptin, intracerebral hemorrhage, prognostic value

## Abstract

The aim of the current study was to examine the prognostic value of copeptin for acute intracerebral hemorrhage (ICH) patients. A total of 120 patients were recruited. The plasma copeptin levels were measured using sandwich immunoassays. The hematoma volume, Glasgow coma scale (GCS) and ICH score were evaluated. The 90-day functional outcomes were measured with the modified Rankin scale (mRS). Copeptin correlated positively with hematoma volume (r=0.61, P=0.000), Hemphill scores (r=0.78, P=0.000) and white blood cell counts (r=0.58, P=0.000), whereas copeptin correlated negatively with GCS scores (r=−0.79, P=0.000). Copeptin levels were also higher in patients with an unfavorable functional outcome at 90 days than in patients with a favorable outcome (4.14±0.87 vs. 3.09±0.30 ng/ml; t=8.001, P=0.00). Monovariate logistic regression analysis results suggest that copeptin is a predictor of the 90-day functional outcomes of ICH patients (OR=3.17, 95% CI 2.01–4.35, P=0.003. Multivariate logistic regression analysis results indicate that copeptin is an independent predictor of the 90-day functional outcomes of ICH patients.

## Introduction

Cerebral hemorrhage is a serious disease that has high disability and mortality rates (1–3). Therefore, an indicator that is closely associated with clinical manifestations, that may be measured quickly and gives a good prediction of the mortality rate, would be beneficial in the early prognosis of cerebral hemorrhage. Currently, it is considered that cerebral hemorrhage-related hematoma formation (4,5), increased bleeding and persistent hypertension are associated with early neurological degeneration and poor prognosis (6).

Arginine vasopressin (AVP), which is one of the most important stress hormones, is a bioactive peptide with nine amino acid residues, that is produced by the hypothalamus (7). However, when AVP is released into the blood in pulse mode, it is extremely unstable with a short biological half-life. Thus, the clinical application of AVP measurement is limited. Copeptin (5 kDa) is the 39-amino acid carboxy-terminal domain of pro-AVP, which includes a leucine-rich core fragment. AVP and copeptin are released equally in the body, thus the direct measurement of AVP may be replaced by the measurement of copeptin (8). Copeptin is important in the maturation, transportation and intracellular processing of AVP and it enables the misfolded monomers to re-fold, to ensure the stability of its biological effects (9).

Copeptin levels are considered to reflect the severity and prognosis of a variety of diseases. Jochberger *et al*(10) compared the serum copeptin levels of 70 healthy individuals with those of 157 intensive care unit (ICU) patients within 24 h of cardiac surgery and identified that the copeptin levels of the ICU group were significantly higher than those of the healthy individuals. Neuhold *et al*(11) performed a 1-year follow-up of 786 patients with various stages of heart failure and revealed that copeptin was the most effective predictor of mortality in New York Heart Association (NYHA) stage II and III patients. Voors *et al*(12) completed a 2-year follow-up of 224 patients and identified that copeptin was an effective prognostic indicator of heart failure following acute myocardial infarction (AMI); it was found to be more effective than the currently accepted indicators, B-type natriuretic peptide (BNP) and amino-terminal B-type natriuretic peptide (NT-proBNP).

In the current study, copeptin was identified to be significantly elevated in the serum of acute cerebral hemorrhage patients and the levels of copeptin increased as the amount of bleeding and cerebral hemorrhage worsened. Logistic regression analysis revealed that copeptin level, Glasgow coma scale (GCS) score and Hemphill score were independent indicators in the evaluation of impaired 90-day nerve function following cerebral hemorrhage.

## Materials and methods

### Patients

A total of 120 patients with acute intracerebral hemorrhage (ICH) were enrolled, including 76 males and 44 females. Sixty healthy individuals were selected as a control group. Prior written and informed consent was obtained from every patient and the study was approved by the ethics review board of Ji Ning Medical University (Shandong, China).

### Clinical evaluation

The severity of ICH was assessed. The hematoma volume was calculated based on a brain computed tomography (CT) scan with ABC/2 methods (A, the maximum diameter of the maximum bleeding layer; B, the diameter of the vertical to A; C, the number of vertical planes multiplied by the thickness of each layer). The GCS and Hemphill ICH scores were calculated. A prognostic evaluation of impaired nerve function was performed according to the Rankin scale score (modified Rankin scale, mRS) after 90 days. A positive outcome was defined as mRS<3 and a poor outcome was defined as mRS≥3.

### Vital signs and biochemical indicators

Fasting blood glucose, white blood cell count and serum sodium were determined. Body temperature, systolic blood pressure and diastolic blood pressure were also measured.

### Enzyme-linked immunosorbent assay (ELISA)

Venous blood (3 ml) was drawn when fasting and centrifuged for 10 min at 3,000 rpm to collect plasma. The serum following centrifugation was stored at −80°C in a refrigerator. Copeptin levels were assessed by ELISA. The ELISA kit was purchased from Phoenix Pharmaceuticals (Burlingame, CA, USA).

### Statistical analysis

Statistics analyses were carried out using SPSS 19.0 software (SPSS Inc., Chicago, IL, USA). Continuous variables were expressed as the mean ± standard deviation. An independent samples t-test was used when comparing two groups. The Chi-square test was applied to enumeration data. The prognostic accuracy of copeptin and other prognostic parameters were analyzed with univariate and multivariate logistic regression models. Spearman's Rho was applied in the correlation analysis. P<0.05 was considered to indicate a statistically significant difference.

## Results

### Clinical characteristics

Among the 120 ICH patients, there were 76 males and 44 females with ages from 32 to 84 years. Bleeding sites differed among the patients: 96 cases were in the basal ganglia region (80%), 12 cases were in a lobe of the brain (10%), 6 cases were in the brain stem (5%) and 6 cases were in the cerebellum (5%). The average plasma copeptin level of the 120 ICH patients was 3.71±0.82 ng/ml compared with 2.82±0.36 ng/ml for the control group of healthy individuals. General clinical data of the ICH patients are listed in Table I.

### Copeptin and other prognostic indicators

Copeptin, Hemphill scores, white blood cell count and Glasgow coma scale (GCS) were determined on admission.

The copeptin level correlated positively with hematoma volume (r=0.61, P=0.000), Hemphill score (r=0.78, P=0.000) and white blood cell count (r=0.58, P=0.000), and negatively with GCS score (r=−0.79, P=0.000). The average level of copeptin (3.71 ng/ml) divided the high and low copeptin groups (Table I). Analyses revealed that hematoma volume, GCS score, Hemphill score, mRS score, systolic blood pressure, white blood cell count and blood glucose differed significantly between the two groups; however, there were no significant differences in the other parameters.

### Copeptin and prognosis of impaired nerve function

After 90 days, prognosis of impaired nerve function was positive in 48 cases (40%) and poor in 72 cases (60%). Plasma copeptin levels were higher at admission in patients with a poor prognosis than in those with a positive prognosis (4.14±0.87 vs. 3.09±0.30 ng/ml, t=8.001, P=0.000). Univariate logistic regression analysis of prognostic indicators of impaired nerve function revealed that copeptin level, white blood cell count, blood glucose, brain hemorrhage and Hemphill score were prognostic indicators (Table II). Multivariate logistic regression analysis revealed that copeptin level, GCS score and Hemphill score were independent prognostic factors for cerebral hemorrhage (Table III).

### Copeptin and mortality rates

During the 90 days of follow-up, 24 patients (20%) succumbed. The copeptin level was higher in the non-survivors than in the survivors (4.97±0.73 vs. 3.40±0.46 ng/ml, t=1.58, P=0.000). Hematoma volume, GCS score, Hemphill score, white blood cell count and blood glucose were significantly different between the non-survivors and survivors (Table IV). No significant differences existed in the other parameters. Univariate logistic regression analysis revealed that the copeptin level, white blood cell count, GCS score, Hemphill score and hematoma volume were valuable for mortality prediction (Table V).

## Discussion

The close correlation between copeptin level and hypothalamic-pituiary-adrenal (HPA) axis activation was the basis of the use of copeptin as a prognostic biomarker (13–16). The current study found that the plasma copeptin level increased in patients with acute ICH. In accordance with the Hemphill score and GCS score, the level of copeptin increased with the severity of ICH. Univariate logistic regression analysis indicated that copeptin level, hematoma volume, GCS score, Hemphill score, white blood cell count and blood glucose are independent prognostic indicators for impaired nerve function, 90 days after ICH.

In the acute stage of ICH, the body enters a state of stress, activated by the HPA axis. Then, corticotropin-releasing hormone is secreted by the hypothalamic paraventricular nucleus. AVP is a hypothalamic-stimulating factor in the HPA axis and has a significant synergistic effect with corticotropin-releasing hormone. Together, they promote the anterior pituitary to release adrenocorticotropic hormone and then stimulate the adrenal cortex to produce cortisol. The level of copeptin increased with the severity of stress and at a far quicker pace than that of cortisol in severe stress conditions (15). Copeptin may receive endogenous information through the thalamus, thus respond to the damage accurately. However, its mechanism remains unclear. Cerebral edema in patients with ICH indicates poor prognosis. The copeptin level may reflect the brain edema formation and its severity, thus helping to determine the existence of cerebral edema, which may be treated with an AVP receptor antagonist.

A previous study demonstrated that higher levels of copeptin in patients with acute progression of chronic obstructive pulmonary disease (COPD) indicated significantly prolonged hospital stay and intensive care unit treatment (17). The Kaplan-Meier survival curve revealed that copeptin levels were significantly higher in patients with poor long-term prognosis. Katan *et al*(15) found that the copeptin level was an independent predictor of brain function and mortality rate three months after acute cerebral infarction and it may improve the accuracy of the National Institute of Health Stroke Score (NIHSS) on neurological damage prognosis and mortality prediction and thus further improve the risk stratification of stroke patients. Urwyler *et al*(18) performed a one-year follow-up based on the above studies and found that copeptin was an independent predictor of brain function and mortality rates one year after cerebral infarction.

Zweifel *et al*(16) determined the copeptin levels of 40 patients with spontaneous ICH and the results revealed the prognostic value of copeptin levels in patients with ICH. However, due to small sample volumes, clinical studies with larger sample numbers are required for further validation. If the prognostic value of copeptin in ICH is verified in large-scale cohort studies, particularly in combination with other prognostic indicators, the risk assessment would be improved. In summary, copeptin is expected to become a new prognostic indicator of ICH.

## Figures and Tables

**Table I.** General clinical data of the patients in this study.

Variable	n=120	High copeptin group (n=58)	Low copeptin group (n=62)	Test value	P-value
Male/total (%)	76 (63%)	32 (55%)	44 (71%)	1.78^a^	0.075
Age (years)	60±14	60±13	61±14	−0.59	0.554
Body temperature (°C)	36.4±0.5	36.5±0.6	36.4±0.4	1.09	0.279
Systolic blood pressure (mmHg)	175±32	181±40	169±22	2.05	0.044
Diastolic blood pressure (mmHg)	103±22	101±22	105±23	−0.92	0.362
White blood cell count (×10^12^/l)	9.98±4.25	11.59±4.82	8.47±3.04	4.25	0.000
Blood glucose (mM)	6.63±2.41	7.50±2.58	5.81±1.96	4.10	0.000
Hyponatremia (mM)	142.38±7.53	144.28±9.99	140.61±3.55	2.67	0.060
Hematoma volume (ml)	57.46±46.76	78.68±49.70	37.60±34.50	5.27	0.000
GCS score	10.60±4.64	6.86±3.62	14.10±2.12	−13.39	0.000
Hemphill score	2.05±1.62	3.24±1.20	0.94±1.08	10.99	0.000
mRS score	3.32±1.95	4.79±1.23	1.94±1.42	11.75	0.000

P-values obtained from t-tests.

a Obtained from Chi-square test. GCS, Glasgow coma scale; mRS, modified Rankin scale.

**Table II.** Univariate logistic regression analysis of prognostic indicators of impaired nerve function.

Prognostic indicators	OR	95% CI	P-value
White blood cell count	1.06	1.01–1.12	0.003
Blood glucose	1.09	1.01–1.18	0.002
Hemphill score	2.19	1.46–3.27	0.000
GCS score	1.62	0.96–2.43	0.001
Hematoma volume	1.02	1.01–1.04	0.000
Copeptin	3.17	2.01–4.35	0.003

OR, odds ratio; CI, confidence interval; GCS, Glasgow coma scale.

**Table III.** Independent prognostic factors for cerebral hemorrhage.

Prognostic indicators	OR	95% CI	P-value
Hemphill score	1.09	0.86–1.27	0.000
GCS score	1.22	0.96–1.43	0.000
Copeptin	1.28	1.05–1.48	0.000

OR, odds ratio; CI, confidence interval; GCS, Glasgow coma scale.

**Table IV.** Prognostic indicators for survivors and non-survivors.

Prognostic indicators	Survivors (n=96)	Non-survivors (n=24)	Test value	P-value
Copeptin (ng/ml)	3.40±0.46	4.97±0.73	1.58	0.000
Hematoma volume (ml)	42.39±31.16	117.72±50.66	75.32	0.000
GCS score	12.25±3.59	4.00±1.25	−8.25	0.000
Hemphill score	1.48±1.26	4.33±0.48	2.85	0.000
Blood glucose (mM)	6.39±2.54	7.59±1.46	1.21	0.030
White blood cell count (×10^12^/l)	8.73±2.70	14.95±5.57	6.21	0.000

GCS, Glasgow coma scale.

**Table V.** Univariate logistic regression analysis of the 90-day mortality rate of patients.

Prognostic indicators	OR	95% CI	P-value
White blood cell count	1.21	1.01–1.43	0.003
GCS score	0.34	0.16–0.72	0.001
Hemphill score	5.14	2.05–8.89	0.000
Hematoma volume	1.11	1.05–1.17	0.000
Copeptin	5.29	3.68–8.03	0.000

OR, odds ratio; CI, confidence interval; GCS, Glasgow coma scale.
